# Cerebral vascular mitochondrial function is dependent on APOE genotype and estrogen status

**DOI:** 10.1002/alz.090516

**Published:** 2025-01-03

**Authors:** Mackenzie N Kehmeier, Alexandra Famiano, Thomas A Leonhardt, Sky J Ferguson, Nabil J Alkayed, Ashley E Walker

**Affiliations:** ^1^ University of Oregon, Eugene, OR USA; ^2^ Oregon Health and Science University, Portland, OR USA

## Abstract

**Background:**

Postmenopausal females who carry an *APOE4* allele are at higher risk of late‐onset Alzheimer’s Disease (LOAD) compared to age‐matched *APOE4* males. Estrogen deficiency predisposes females to an increased risk of vascular, cognitive and metabolic impairments. Estrogen and *APOE* genotype are known to impact metabolic and mitochondrial function in the brain, but their effects on cerebral vessels are unknown. Thus, the purpose of this study was to determine the interaction between *APOE* genotype and estrogen deficiency in relation to cerebrovascular mitochondrial function.

**Method:**

Young female homozygous *APOE3* and *APOE4* mice (n = 6‐8 per group; 6 months old) fed a high‐fat diet were ovariectomized (“ovx”), ovariectomized and supplemented with 17β‐estradiol (0.36 mg, 60‐day release, “estradiol”), or left intact (“sham”). At 2 months after ovariectomy, a glucose tolerance test (GTT) was performed, then cerebral arteries and arterioles were dissected, incubated in saponin, then assessed for mitochondrial respiration in response to substrates probing carbohydrate metabolism (Oroboros). Data are presented as mean±SEM.

**Result:**

There was an interaction between *APOE* genotype and ovx/estradiol status in relation to cerebrovascular complex I (CI)‐ (p = 0.04) and complex II (CII)‐coupled respiration (p = 0.04). Additionally, there was a significant effect of genotype, such that vessels from *APOE3* mice had greater CI‐coupled respiration than vessels from *APOE4* mice (p = 0.02). When examining differences between *APOE3* groups, *APOE3*‐estradiol mice had a 53% greater CI‐coupled respiration than *APOE3*‐sham mice (15.4±3.4 vs. 7.2±1.2 pmol/(s*mg), p = 0.03) and a 60% greater CI‐coupled respiration than *APOE3*‐ovx mice (6.2±1.6 pmol/(s*mg), p = 0.007). The *APOE3*‐estradiol group also had greater CI+CII‐coupled respiration compared to both *APOE3*‐sham (50.8±7.1 vs. 27.3±1.7 pmol/(s*mg), p = 0.006) and ovx groups (28.0±5.7 pmol/(s*mg), p = 0.009). Interestingly, CI and CI+CII coupled respiration did not differ between sham, ovx, and estradiol in *APOE4* mice (p>0.05). Maximal uncoupled respiration was greater in the *APOE3* mice than *APOE4* mice (p = 0.01). Whole‐body glucose tolerance did not differ between groups (all p>0.05).

**Conclusion:**

Overall, these results indicate that *APOE* genotype modulates the impact of estrogen on the cerebrovasculature. We found that 17β‐estradiol enhances cerebrovascular mitochondrial function in *APOE3* mice but not *APOE4* mice. The results suggest that estradiol supplementation may have more therapeutic benefit for *APOE4* non‐carriers.

